# Iron restriction

**DOI:** 10.1093/emph/eov011

**Published:** 2015-06-14

**Authors:** John H. McCullough

**Affiliations:** Department of Anthropology, Durham University, DH1 3LE Durham, UK

## IRON RESTRICTION

**Figure 1.**
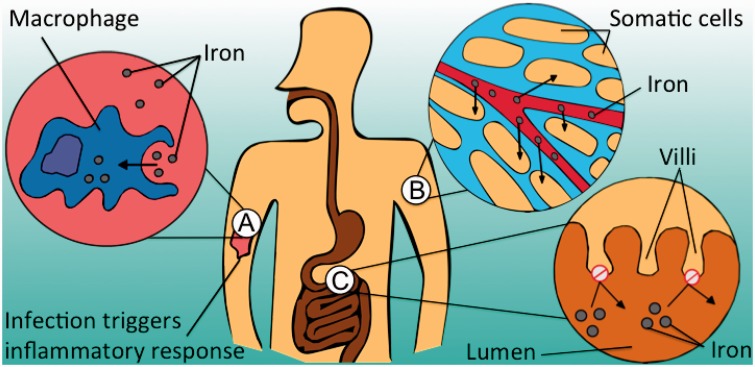
Mechanisms of iron restriction [2]. (**A**) Macrophage absorption and storage of free iron at infection site, (**B**) Redistribution of iron from blood to somatic cells, (**C**) Reduction in gastrointestinal absorption of iron

Iron restriction is the body’s removal of freely available iron from blood and extracellular fluid, as part of the systemic inflammatory response [1]. This process is mediated by hepcidin, which ‘locks’ iron into somatic cells by down-regulating the iron exporter protein ferroportin [2]. Iron accumulates within macrophages, hepatocytes and gut enterocytes, but cannot enter the circulation [1, 2] (see Fig. 1). This reduction in available iron can inhibit erythropoiesis, causing anaemia of inflammation [1]. Though previously assumed to be detrimental to health, a growing body of evidence suggests that iron restriction represents an adaptive host-defence against infection [3].

## EVOLUTIONARY PERSPECTIVES

Iron is essential for the growth and proliferation of almost all microorganisms including bacteria, viruses and protozoa [2]. Many pathogens release chelating agents and scavenge iron-rich proteins from their host [2]. Iron restriction limits microbial access to iron and is likely to be an adaptive product of host-pathogen coevolution, which has been conserved because it reduces infection-related mortality [3].

In support of this hypothesis, iron overload is associated with poor clinical outcomes in TB [4] and HIV [5], but iron deficiency is protective against malaria [6]. Patients with haemochromatosis (a condition of iron excess) are particularly susceptible to infection, even by organisms that are usually benign [7]. Recent genetic analyses across primate species reveal evidence of an ‘evolutionary arms race’ between transferrin (an iron-transport protein) and iron-scavenging bacteria [3]. Finally, evidence suggests hepcidin evolved from an antimicrobial peptide to acquire iron-regulatory functions during vertebrate evolution [8].

## FUTURE CLINICAL IMPLICATIONS

In certain situations iron supplementation may be harmful. A randomized controlled trial (*n* = 24 000) showed that iron and folic acid supplements increase the risk of malarial infection and mortality in children with normal iron status, but not in iron-deficient children [9]. This contentious result demands further investigation into the potential risks of iron therapy.

An evolutionary approach to iron raises further questions. Should we suspend iron therapy when treating infection? Does dietary iron intake alter susceptibility to infection? Could iron chelating agents [2] yield a new class of antibiotic?
